# EMD386088 (5 mg/kg) has no effect on latent inhibition shown to both light and noise stimuli

**DOI:** 10.1177/0269881119882855

**Published:** 2019-10-17

**Authors:** Helen Joan Cassaday, Karen Elizabeth Thur

**Affiliations:** School of Psychology, University of Nottingham, Nottingham, UK

**Keywords:** Fear conditioning, latent inhibition, 5-hydroxytyptamine, EMD386088

## Abstract

Activation of 5-hydroxytyptamine_6_ (5-HT_6_) receptors stimulates attentional switching and 5-HT_6_ receptor antagonists are putative drugs for psychosis. Latent inhibition (LI) provides a pre-clinical model of attentional switching and ‘antipsychotic-like’ action and is known to be modulated by 5-hydroxytyptamine. In the present study, LI was shown in a fear conditioning procedure that measured suppression of drinking after conditioning with footshock. In two experiments (each *n* = 48) it was shown that pre-exposure to both light- and noise-conditioned stimuli reduced conditioned suppression relative to the corresponding non-pre-exposed control. However, counter to prediction, LI was intact after treatment with the 5-HT_6_ agonist EMD386088 (5 mg/kg).

## Introduction

Amongst the multiplicity of 5-hydroxytyptamine receptor sub-types, the 5-hydroxytyptamine_6_ (5-HT_6_) receptor in particular is located in brain regions involved in learning and memory (Fone, 2008; Ivachtchenko et al., 2016). Consistent with this receptor distribution, 5-HT_6_ receptor antagonists can improve learning and memory in a variety of procedures (Fone, 2008; Ivachtchenko et al., 2016). However, the evidence for their efficacy in animal models for schizophrenia is limited (Gravius et al., 2001).

Latent inhibition (LI) provides a pre-clinical model of attentional switching to test drugs for psychosis (Nelson et al., 2011; Weiner, 1990). Specifically, LI refers to the reduction in associative learning produced by pre-exposure to the intended conditioned stimulus (CS), relative to a non-pre-exposed group for which the CS is novel. With experimental parameters set to produce weak LI in controls, there was no evidence for enhancement of LI following treatment with 5-HT_6_ antagonists (Leng et al., 2003). However, a variety of serotonergic manipulations have well-documented effects on LI (Weiner, 1990) and the effects of 5-HT_6_ agonists have not been reported.

In the present study, a similar fear conditioning procedure (suppression of drinking after conditioning with footshock) was used to test the prediction that treatment with the 5-HT_6_ agonist EMD386088 would reduce LI, by restoring conditioning to the pre-exposed stimuli. EMD386088 was administered at 5 mg/kg, the dose previously identified to attenuate prior learning in a similar fear conditioning procedure (supplemental material). This dose also restored fear conditioning that was attenuated because of cholinergic hypoactivity (Woods et al., 2012).

## Methods

For each experiment, 48 experimentally naïve adult male Wistar rats (Charles River, UK; average start weight 220 g) were caged in pairs on a 12:12 h light/dark cycle with *ad libitum* food. All procedures were carried out in accordance with the United Kingdom (UK) Animals Scientific Procedures Act 1986, Project Licence number: PPL40/3163 and following an established LI procedure (Nelson et al., 2011).

Water deprivation was used to motivate licking in a conditioned suppression of drinking procedure, conducted within six automated conditioning boxes (Cambridge Cognition, Cambridge, UK). In Experiment 1, a flashing light of overall 5 s duration served as the CS for the control group of rats and was first presented without consequence (×30) in the pre-exposed (PE) groups. In Experiment 2, a 5 s mixed frequency noise set at 85 dB served as CS for the control group and was first presented without consequence (×30) in the PE groups. In both experiments, a scrambled footshock of 1 s duration and 1 mA intensity provided the unconditioned stimulus (Nelson et al., 2011). Both experiments used a 4-day procedure for pre-exposure, conditioning, reshaping and test (Nelson et al., 2011).

EMD386088 HCl (Tocris, UK) was dissolved in saline at 5 mg/mL for injection (i.p.) at 1 mL/kg to administer a dose of 5 mg/kg. Drug or saline (1 mL/kg) control injections were administered 30 min prior to the pre-exposure and conditioning stages of the LI procedure.

Associative learning and LI thereof was measured as suppression ratios.

## Results

In both experiments, the baseline licking scores seen pre-conditioning confirmed that the rats were well-matched across their experimental allocations (Table 1). As might be expected, after conditioning, the latencies to drink in the boxes were longer and the rats drank less, reflecting fear conditioning to context measured on the reshaping day. However, there was no effect of EMD386088 on fear conditioning to context in either experiment.

**Table 1.** Mean lick latencies and numbers of licks (±SEM) in experiments 1 and 2 (*n =* 24/drug group/experiment). Data were analysed with between subjects factors of drug (saline, EMD386088) and the allocated behavioural condition (pre-exposed, non-pre-exposed). The *p-*values shown are for the main effect of drug and for the interaction term. There were 5 days of pre-conditioning during which rats became accustomed to drinking in the boxes. The data from the 5th day are shown for direct comparison with the reshaping day which followed pre-exposure and conditioning under drug.

	Measure	Saline (*n* = 24 × 2)	EMD386088 5mg/kg (*n* = 24 × 2)	Statistics for main effect of drug (*p*)	Statistics for drug × pre-exposure interaction (*p*)
Experiment 1					
Pre-conditioning	latency	5.21 (1.29)	6.25 (1.47)	0.60	0.95
	min 1 lick	280.92 (8.28)	276.50 (10.99)	0.75	0.25
	total licks	1860.38 (101.06)	1906.46 (98.11)	0.75	0.98
Reshaping	latency	162.79 (49.92)	108.71 (27.94)	0.36	0.92
	min 1 lick	227.17 (17.21)	200.58 (21.14)	0.35	0.89
	total licks	1676.75 (119.95)	1627.21 (126.72)	0.78	0.99
Experiment 2					
Pre-conditioning	latency	7.38 (1.85)	6.92 (2.42)	0.88	0.61
	min 1 lick	284.42 (14.09)	281.79 (13.56)	0.89	0.36
	total licks	1931.58 (114.76)	1862.75 (96.36)	0.65	0.34
Reshaping	latency	23.13 (4.41)	66.83 (22.94)	0.07	0.57
	min 1 lick	236.46 (18.80)	197.92 (22.12)	0.19	0.98
	total licks	1819.08 (118.80)	1712.25 (73.91)	0.45	0.54

Note: SEM = standard error of the mean.

### Effects of EMD386088 on latent inhibition with a light CS

**Figure 1.**
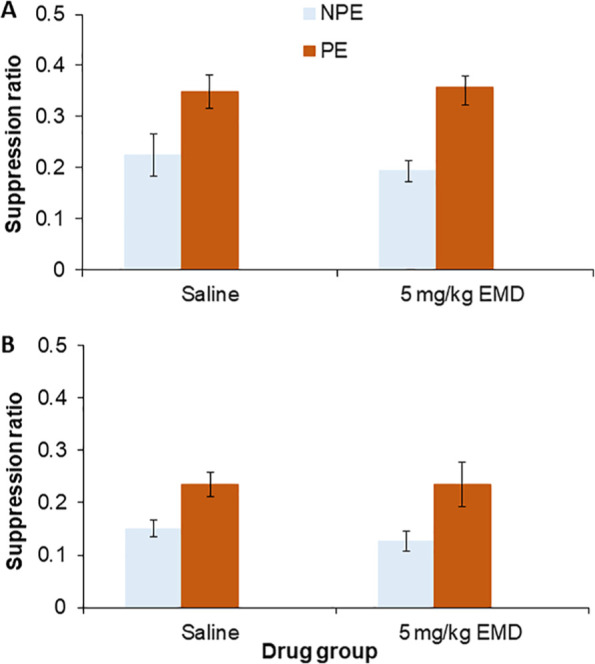
(A) Mean suppression ratio (±SEM) to the light for control (light blue) and pre-exposed (PE; dark orange) groups following treatment with saline or 5 mg/kg EMD386088 in Experiment 1 (*n =* 47). (B) Mean suppression ratio (±SEM) to the noise for control (light blue) and pre-exposed (dark orange) groups following treatment with saline or 5 mg/kg EMD386088 in Experiment 2 (*n =* 48). Note: SEM = standard error of the mean.

In the key conditioned suppression tests (Figure 1A) data were lost from one rat due to equipment failure. There was a main effect of conditioning group (*F*(1,43) = 18.41, *p* < 0.001). Thus LI was demonstrated with the light CS. However, there was no effect of drug, either overall or in interaction with pre-exposure (maximum *F*(1,43) = 0.352).

### Effects of EMD386088 on latent inhibition with a noise CS

Analysis of variance showed a main effect of conditioning group (*F*(1,44) = 12.46, *p* = 0.001). Thus LI was also demonstrated with the noise CS (Figure 1B). However, there was no effect of drug, either overall or in interaction with pre-exposure (maximum *F*(1,44) = 0.21).

## Conclusion

Contrary to prediction, there was no indication of any effect of 5 mg/kg EMD386088 on LI. It is a limitation of the present study that further doses were not examined. However, the dose selected for use has previously been reported effective (Woods et al., 2012). The present study used 30 pre-exposures to the subsequent CS, resulting in robust LI irrespective of whether this stimulus was light (Experiment 1) or noise (Experiment 2). Since in common with 5-HT_6_ antagonists, 5-HT_6_ agonists can show paradoxical pro-cognitive effects (Fone, 2008) and EMD386088 is a partial agonist with an irregular dose-response (Jastrzębska-Więsek et al., 2013), it remains possible that LI enhancement under EMD386088 could be revealed under conditions of fewer pre-exposures, drug-induced impairment, or at a different dose.

## Supplemental Material

Suppl_file_to_show_EMD_effect – Supplemental material for EMD386088 (5 mg/kg) has no effect on latent inhibition shown to both light and noise stimuli Supplemental material, Suppl_file_to_show_EMD_effect for EMD386088 (5 mg/kg) has no effect on latent inhibition shown to both light and noise stimuli by Helen Joan Cassaday and Karen Elizabeth Thur in Journal of Psychopharmacology
