# Supplementary material for: EMD386088 (5 mg/kg) has no effect on latent inhibition shown to both light and noise stimuli
Source: J Psychopharmacol. 2019 Oct 17;34(1):145–7. doi: 10.1177/0269881119882855 (PMC6947809; doi:10.1177/0269881119882855)
Supplement: Suppl_file_to_show_EMD_effect – Supplemental material for EMD386088 (5 mg/kg) has no effect on latent inhibition shown to both light and noise stimuli [file Suppl_file_to_show_EMD_effect.docx]

**Supplementary data obtained using EMD386088 5mg/kg**

Overshadowing procedures use the relative intensity of competing CSs to manipulate associability: normally in compound conditioning a relatively more intense CS acquires associative strength at the expense of a relatively less intense CS. Such an overshadowing procedure was run at the same time as one of the LI studies reported, in the same apparatus with otherwise matched lick suppression procedures. However, unlike LI, the successful demonstration of overshadowing required the use of 2 conditioning sessions.

As in the LI study, EMD 386088 HCl (Tocris, UK) was dissolved in saline at 5mg/ml for injection (i.p.) at 1ml/kg to administer a dose of 5mg/kg. EMD386088 5mg/kg did not affect conditioning to the cue which was attenuated because of cue competition. This lack of effect on the selection of cues for learning is broadly consistent with the lack of effect on LI reported here. However, EMD386088 5mg/kg was not entirely effective in that, consistent with impaired consolidation, there was evidence that the effects of prior conditioning were reduced.

Rats reconditioned under the selected 5mg/kg dose of EMD386088 showed less evidence of prior learning compared to their saline-injected counterparts. Statistically, at the reconditioning stage of the procedure, there was no effect of conditioning group [F(1,43)=0.888] or conditioning group by drug interaction [F(1,43)=3.276, p=0.077]. However, there was a clear main effect of drug [F(1,43)=21.164, p<0.001] which took the form of overall less suppression under EMD386088 (Figure 1S).

----- Figure 1S about here -----


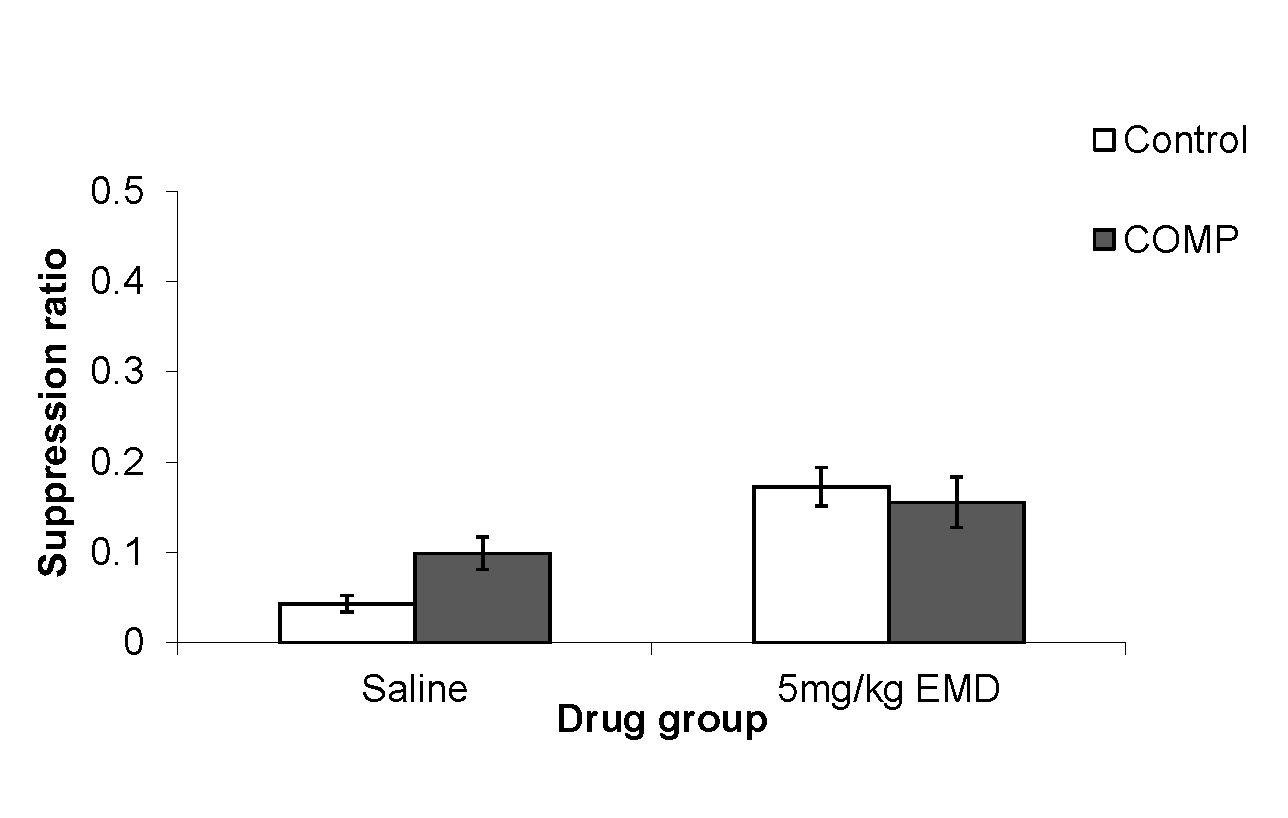


**Figure 1S:** Mean suppression ratio (±S.E.M.) to a reconditioned noise CS for control (white bars) and compound conditioned (COMP; dark grey bars) groups following treatment with saline or 5 mg/kg EMD 386088.

Although the overshadowing experiment was not designed to examine consolidation, the drug effect demonstrated at reconditioning means that rats treated with EMD86088 showed a reduced effect of prior conditioning relative to their saline-treated counterparts, consistent with impaired consolidation of associative learning.

Similarly, EMD386088 5mg/kg has been found to impair the consolidation of simple associative learning measured in an appetitive autoshaping procedure. Doses in the range of 1-10mg/kg were tested in this previous study and 5mg/kg was identified as the behaviourally effective dose (Meneses et al. Behav Brain Res 2008;195:112-9).

In contrast in the LI study, there was no evidence for any failure to consolidate whatever is learned at the pre-exposure stage of the procedure.
